# High water temperature significantly influences swimming performance of New Zealand migratory species

**DOI:** 10.1093/conphys/coae047

**Published:** 2024-07-31

**Authors:** Rachel M B Crawford, Eleanor M Gee, Deborah W E Dupont, Brendan J Hicks, Paul A Franklin

**Affiliations:** School of Science, Environmental Research Institute, The University of Waikato, Room E.2.20, E Block, Gate 8, Hillcrest Road, Hamilton, 3216, New Zealand; National Institute of Water and Atmospheric Research, Gate 10, Silverdale Road, Hillcrest, Hamilton, 3216, New Zealand; National Institute of Water and Atmospheric Research, Gate 10, Silverdale Road, Hillcrest, Hamilton, 3216, New Zealand; Waikato Regional Council, 160 Ward Street, Hamilton Central, Hamilton, 3204New Zealand; National Institute of Water and Atmospheric Research, Gate 10, Silverdale Road, Hillcrest, Hamilton, 3216, New Zealand; School of Science, Environmental Research Institute, The University of Waikato, Room E.2.20, E Block, Gate 8, Hillcrest Road, Hamilton, 3216, New Zealand; Morphum Environmental Ltd, 65 Victoria Street, Hamilton, 3204New Zealand; National Institute of Water and Atmospheric Research, Gate 10, Silverdale Road, Hillcrest, Hamilton, 3216, New Zealand

**Keywords:** Critical swimming speed, fish passage, fish swimming, metabolic rate, temperature, Ucrit

## Abstract

Anthropogenic structures in freshwater systems pose a significant threat by fragmenting habitats. Effective fish passage solutions must consider how environmental changes introduce variability into swimming performance. As temperature is considered the most important external factor influencing fish physiology, it is especially important to consider its effects on fish swimming performance. Even minor alterations in water properties, such as temperature and velocity, can profoundly affect fish metabolic demands, foraging behaviours, fitness and, consequently, swimming performance and passage success. In this study, we investigated the impact of varying water temperatures on the critical swimming speeds of four migratory New Zealand species. Our findings revealed a significant reduction in critical swimming speeds at higher water temperatures (26°C) compared to lower ones (8 and 15°C) for three out of four species (*Galaxias maculatus*, *Galaxias brevipinnis* and *Gobiomorphus cotidianus*). In contrast, *Galaxias fasciatus* exhibited no significant temperature-related changes in swimming performance, suggesting species-specific responses to temperature. The cold temperature treatment did not impact swimming performance for any of the studied species. As high water temperatures significantly reduce fish swimming performance, it is important to ensure that fish passage solutions are designed to accommodate a range of temperature changes, including spatial and temporal changes, ranging from diel to decadal fluctuations. Our research underscores the importance of incorporating temperature effects into fish passage models for habitat restoration, connectivity initiatives, and freshwater fish conservation. The influence of temperature on fish swimming performance can alter migration patterns and population dynamics, highlighting the need for adaptive conservation strategies. To ensure the resilience of freshwater ecosystems it is important to account for the impact of temperature on fish swimming performance, particularly in the context of a changing climate.

## Teaser

Our study examined how temperature differences affect critical swimming speeds of four migratory species. Higher temperatures (26°C) significantly reduced swimming speeds for three species, emphasizing the need for fish passage solutions that consider temperature fluctuations. This is crucial for habitat restoration and freshwater fish preservation, especially in a changing climate.

## Abbreviations

ANCOVA, Analysis of Covariance
bl s^−1^, body lengths per second
βO_2_, solubility of oxygen (ml L^−1^)
*U_crit_*, critical swimming speed (bl s^−1^)
M_fish_, mass of the fish (g)
MMR, maximum metabolic rate (mg O_2_ g^−1^ h^−1^)
ΔO_2_, percent change in air saturation per hour
SMR, standard metabolic rate (mg O_2_ g^−1^ h^−1^)
Δt, time interval (seconds)
*t_f_*, time the fish swam at the highest speed (seconds)
ΔU, incremental change in water speed (bl s^−1^) U_f-1_, penultimate water speed (bl s^−1^)
*V*, volume (L)

## Introduction

Anthropogenic instream structures fragment habitats, posing a significant threat to freshwater ecosystems (Castro-Santos *et al.,* 2009; Bunt *et al.,* 2012; Fangue *et al.,* 2015; Franklin and Gee, 2019). This fragmentation isolates fish populations and obstructs diadromous fish migration, limiting dispersal and reducing species richness (Nicola *et al.,* 1996; Lucas and Baras, 2001; Daufresne and Boët, 2007; Gough *et al.,* 2012; Jellyman and Harding, 2012; Neachell, 2014; Radinger and Wolter, 2014; Silva *et al.,* 2018; Wilkes *et al.,* 2019). These barriers to migration lead to global diadromous fish loss and degrade freshwater ecosystems (Bunt *et al.,* 2012; Fangue *et al.,* 2015; Franklin and Gee, 2019).

Restoring river connectivity is a key restoration goal for freshwater ecosystems (Thieme *et al.,* 2023; Franklin *et al.*, 2024). Approaches to designing more effective fish passage solutions must consider not only inter- and intra-species variability in behaviour and capabilities (Crawford *et al.,* In Review), but also how changes in the environment introduce variability into swimming performance (Jones *et al.*, 2021). Even subtle changes in water properties, such as temperature and velocity, can have profound effects on fish metabolic demands, foraging behaviours and overall fitness (Green and Fisher, 2004; Fangue *et al.,* 2015) and subsequently, fish swimming performance and passage success.

Water temperature plays a crucial role for fish by regulating physiological and cellular processes (Brett and Brett, 1971; Schurmann and Steffensen, 1997; Claireaux and Lagardère, 1999). Water temperature exerts a direct influence on fish metabolism, as it increases the standard metabolic rate (SMR) and maximum metabolic rate (MMR), impacting tasks such as predator avoidance, foraging and swimming (Brett, 1964; Claireaux and Lagardère, 1999; Sandblom *et al.,* 2014). Although increasing water temperatures have been shown to exponentially increase standard metabolic rate, maximum metabolic rate may either continue to increase, plateau or even decrease depending on fish species and life stage (McKenzie and Claireaux, 2010; Norin and Clark, 2016). Additionally, changes in water temperature influence water oxygen saturation levels and oxygen consumption by organisms, further affecting fish swimming abilities (Schurmann and Steffensen, 1997). The effects of water temperature on fish swimming vary by species, with some species more susceptible to colder temperatures and others warmer temperatures, often depending on whether they are temperate, tropical or cold-water species (Johnston and Dunn, 1987; Fangue *et al.,* 2015; Parisi *et al.,* 2020; Muhawenimana *et al.,* 2021). Water temperature also serves as an ecological factor influencing niche partitioning, determining the habitat preferences and distributional limits for fish populations (Magnuson *et al.,* 1979; Richardson *et al.,* 1994).

Freshwater systems are highly dynamic and are characterized by thermal heterogeneity often driven by atmospheric conditions, topography, stream discharge and streambed characteristics (Caissie, 2006; Pander *et al.,* 2024). These drivers are influenced by various environmental factors such as transitions from sea to freshwater environments, temperature differentials where tributaries meet larger systems and differences in riparian shade versus direct solar radiation (Caissie, 2006; Tague *et al.,* 2007). Additionally, small-scale temperature changes occur due to groundwater inputs, including hyporheic flows, springs and seeps (Hatch *et al.,* 2006). Steep topography, such as stream slope, has been linked to cooling water, as it facilitates increased mixing between surface water and interstitial water, and increased evaporative cooling (Pander *et al.,* 2024).

Anthropogenic activities further contribute to stream thermal heterogeneity (Caissie, 2006). Water abstraction and removal practices can elevate river temperatures beyond normal levels (Booker & Whitehead, 2022) and in urban systems, stormwater runoff from impervious surfaces on heat islands leads to dramatic increases in stream temperature not found in forested streams (Somers *et al.,* 2013). Land use changes resulting in differences in riparian canopy cover significantly influences water temperature, with shaded sections of stream having lower water temperatures than sections with open canopy (Somers *et al.,* 2013; Pander *et al.,* 2024). Hot water released from power stations and cold water released from dams are significant contributors to water pollution in riverine systems (Parisi *et al.,* 2020). Impoundments exacerbate temperature stratification in water columns that are typically well mixed, resulting in the loss of suitable fish habitat (Parasiewicz *et al.*, 2023). Thermal heterogeneity affects fish behaviour and habitat use, with fish known to seek out cold water refugia when stream temperatures exceed their thermal optima impacting fish during migration (Sutton *et al.,* 2007).

New Zealand’s diverse geographic features, varied landscapes and significant latitudinal gradients contribute to a wide range of water temperatures in its freshwater systems (Warrick *et al.,* 2001). Amphidromous species are prevalent within New Zealand’s native fish communities, beginning upstream migration as small-bodied juveniles. These species undertake extensive journeys inland and to high elevations during migration, experiencing a wide range of temperatures (McDowall, 1990; Franklin and Gee, 2019). During upstream migration, water temperatures across the country can span from <1°C to >26°C, with average water temperatures ranging from 9 to 18°C (Mosley, 1982; New Zealand National Water Quality Monitoring Network, 2024; Table 1). New Zealand’s fish are susceptible to acute temperature changes during their migration periods, arising from both natural and anthropogenic factors. For example increased land use for agriculture and forestry has resulted in more extreme water temperature fluctuations and seasonal water temperature variation (Quinn *et al.,* 1992; Harding *et al.,* 2000; Collier and Bowman, 2003; Larned *et al.,* 2020). Furthermore, the prevalence of thermal power stations on New Zealand’s large rivers are known to create hot water plumes, heating river temperatures >10°C >1 km downstream from the point source (e.g. Wairakei Geothermal Power Station, Waikato River; Baker *et al.,* 2021). These natural and anthropogenic factors result in not only seasonal and spatial changes in water temperature, but also diurnal and sub-daily fluctuations. Despite the critical role of water temperature in fish physiology and ecological dynamics, limited research has been conducted on the impacts of temperature on fish swimming ability in New Zealand.

**Table 1:** Seasonal water temperatures (°C) across New Zealand, including minimum, maximum and average temperatures from 1989 to 2024, from New Zealand National Water Quality Monitoring Network

**Season**	**Mean**	**Minimum**	**Maximum**	**SD**
Spring	12.82	3.50	26.80	3.25
Summer	18.14	7.10	31.40	3.43
Autumn	14.37	4.50	25.70	3.42
Winter	9.07	0.40	21.00	2.54

The primary objective of this research is to investigate the influence of acute water temperature changes on the swimming performance of native diadromous New Zealand fish species. We conducted critical swimming speed measurements on *Galaxias maculatus* (inanga), *Galaxias fasciatus* (banded kokopu), *Galaxias brevipinnis* (koaro) and *Gobiomorphus cotidianus* (common bully), at three distinct water temperatures, 8, 15 and 26°C, which span the range of average summer temperatures in New Zealand’s rivers. These four species were chosen as they all have widespread populations across the country, ranging in both latitude and elevation, and varying landscapes. These diadromous species must all migrate from the sea through the lowlands, exposing them to a range of water temperatures on their migration upstream.

By investigating how these acute water temperature differences affect fish swimming abilities, we can develop fish passage design strategies that consider the thermal challenges fish face in their habitats. It is important to design fish passage solutions that encompass the range of water temperatures fish communities will encounter now and into the future to ensure continuous accessibility for upstream migration (Birnie-Gauvin *et al.,* 2019; Lynch *et al.,* 2023). As climate change is expected to amplify temperature fluctuations, it is especially important to understand the ecological implications of acute temperature effects for future-proofing conservation and management strategies (sensu Lynch *et al.,* 2023).

## Materials and Methods

### Fish collection and handling

Ethics approval and collection permits were secured from the relevant authorities (Ministry for Primary Industries Special Permit SP666–4; approval from NIWA Animal Ethics Committee, AEC204).

Juvenile *G. maculatus* were collected in spring using fyke nets at the mouth of New Zealand’s Rangitaiki River (37°54′34.6” S 176°52′53.0″ E). Juvenile *G. fasciatus* were collected in spring using fyke nets from the Lilleburn Stream in the Hunua Ranges (37° 04′ 55.6” S 175° 10′ 02.9″ E) and Waitawhara Stream in New Zealand’s Glen Afton (37° 36′ 20.6” S 175° 02′ 12.8″ E). Juvenile *G. brevipinnis* were also collected in spring using fyke nets from the Lilleburn Stream in the Hunua Ranges (37° 04′ 55.6” S 175° 10′ 02.9″ E). Juvenile *G. cotidianus* were collected in autumn using seine netting from Waiotapu Stream (38°25′11.4” S 176°20′43.7″ E). Whilst water temperature was not measured at time of fish collection, maximum daily air temperature was between 10 and 15°C across all fish collections.

All fish were transported to NIWA Hamilton laboratory in well oxygenated stream water. Upon arrival, the fish were acclimated to the holding tank’s temperature (15°C) and placed in 60 L quarantine tanks containing water with 6 ppt salinity as a precaution against disease. After a week, they were moved to 60-l tanks with dechlorinated water connected to a recirculating water system. Water temperature was maintained at 15 ± 0.5°C, and fish were kept on a 12-h day/12-h night cycle. Fish were fed bloodworms every second day starting once they were transferred into holding tanks and were subjected to a 24-h fast prior to trials to ensure a post-absorptive state. Regular monitoring of ammonia and pH levels was carried out, and water changes were conducted to maintain water quality if ammonia concentrations exceeded 0.25 mg l^−1^.

### Temperature acclimation

The trial water temperatures selected for this study were 8, 15, and 26°C as they are environmentally relevant temperatures representing the typical range found in New Zealand stream systems (New Zealand National Water Quality Monitoring Network, 2024). Neither 8 nor 26°C falls within the lethal water temperature range for the fish under investigation (Richardson *et al.,* 1994).

To avoid the impacts of sudden changes in water temperature, the fish were acclimated to the trial temperature prior to testing. Once fish were initially acclimated to the laboratory as described above, the water temperature in the fish holding tanks was incrementally increased or decreased by 2°C every day until the desired water temperature was reached. Fish were then held for a minimum of 48 h at the trial temperature before placing them in the swim tunnel (which was set to the trial temperature) and undertaking trials. Once acclimated to the trial temperature, fish in the 8 and 26°C temperature treatments were held for a maximum of 3 days before testing. Fish in the ambient treatment of 15°C were held for a maximum of 15 days. An analysis of covariance showed that there was no significant effect of treatment holding time on critical swimming speed (*P* < 0.05), for each species*,* where swimming speed was the response variable and fish length, water temperature and holding time were predictor variables.

### Experimental setup

The experimental setup and procedure mirrored that of Crawford *et al.* (2023). Critical swimming speed trials were conducted using a 10-l swim tunnel (Steffensen-type swim tunnel from Loligo Systems). Water speed within the tunnel was controlled using Autoresp v2.3.0 (Loligo Systems), which also compensated for the solid blocking effect (Kline *et al.,* 2015). Throughout the trials, Water temperature in the swim tunnel was kept at 8 ± 0.5, 15 ± 0.5, or 26 ± 0.5°C depending on temperature treatment. Dissolved oxygen percent saturation within the swim tunnel was maintained at 92.3 ± 1%.

### Experimental procedure

At the start of each trial, a single fish was measured (total length) and then placed into the swim tunnel and acclimated for 30 min at a water speed of 0.5 body lengths per second (bl s^−1^). Trials concluded when a fish displayed signs of fatigue, remaining stationary against the mesh at the back of the tunnel for a continuous period of 3 s or for a total of 10 s within a 30-s window. After a trial, fish were given a 20-min rest period to aid recovery before being returned to the holding tank. Each trial was conducted independently, with no reuse of fish for subsequent trials.

Modified critical swimming speed (*U_crit_*) trials were conducted to accommodate the distinct behaviours of the species under study (Crawford *et al.,* 2023). For pelagic swimming species (*G. maculatus*), water speed was increased by 1 bl s^−1^ every 300 s. For station-holding and benthic-associated species (e.g. *G. cotidianus, G. fasciatus* and *G. brevipinnis*), water speed was raised by 1 bl s^−1^ every 10 s. The U*_crit_* test was conducted at each of the water temperature treatments (8, 15 and 26°C) for each species. Juveniles of all species were used with total length <7 cm for *G. maculatus*, *G. fasciatus* and *G. brevipinnis* (Table 2). Total length of *G. cotidianus* was <4.7 cm (Table 2).

**Table 2:** Species data for temperature trials, including the number of species tested at each temperature, their mean total lengths and standard deviation

**Species**	**Temperature (°C)**	**N**	**Mean Length (cm)**	**SD (±)**
***G. maculatus***	8	17	4.6	0.48
	15	19	4.4	0.38
	26	18	4.2	0.21
***G. fasciatus***	8	19	4.4	0.6
	15	20	4.3	0.53
	26	20	4.2	0.28
***G. brevipinnis***	8	20	5.8	0.54
	15	20	5.2	0.6
	26	19	5.7	0.49
***G. cotidianus***	8	20	3.3	0.35
	15	20	3.2	0.33
	26	19	3.6	0.44

The critical swimming speed (relative to fish length) of each fish was calculated using the following equation from Brett (1964):


$$ {U}_{crit}={U}_{f-1}+\Delta U\left(\frac{t_f}{\Delta t}\right) $$

Where *U_f-1_* represents the penultimate water speed the fish was subjected to (bl s^_1^), Δ*U* represents the incremental change in water speed (i.e. 1 bl s^−1^), *t_f_* is the time the fish swam at the highest speed in seconds (0 ≤ *t_f_* < *Δt*), *Δt* represents the time interval in seconds (i.e. 300 or 10 s depending on species). Swimming speed units were recorded in body lengths per second.

To test the effects of water temperature on fish critical swimming speed for each individual species, an analysis of covariance (ANCOVA) was used. Water temperature was used as a categorical predictor and fish length as a continuous predictor variable. Swimming speed in body lengths per second (the value of U*_crit_*) was used as the response variable. To assess model fit, a Likelihood Ratio Test was used, starting with a fully saturated model including interactions. The interaction between body length and water temperature treatment was not statistically significant for any species and was removed from the models. For each species, length was not statistically significant, but was kept in the models as length is known to influence *U_crit_* (Beamish, 1978; Cano-Barbacil *et al.,* 2020). A *post hoc* Tukey Honestly Significant Different (HSD) test was used to determine, per species, which temperature treatments had statistically different critical swimming speeds from one another. All statistical analyses were performed using R statistical computing software v4.0.3 (Wickham, 2016; R Core Team, 2020).

Due to logistical constraints, we focused our further metabolic experiments on *G. maculatus*. This species is commonly used as a benchmark for designing fish passes in New Zealand because of its slow swimming speeds compared to other migratory species (Crawford *et al.,* In Review). This species was also chosen because of its cultural and commercial significance in New Zealand, its widespread distribution in the temperate Southern Hemisphere and its tendency to experience higher temperatures due to its adult habitat being in low-lying areas closer to the coast (McDowall, 1990). Maximum metabolic rate of *G. maculatus* was recorded immediately after termination of *U_crit_* trials at 15 and 26°C. Further methodology and results from this set of experiments can be found in supplementary materials (Table S1, Fig. S1).

## Results

### Temperature effects

**Figure 1:**
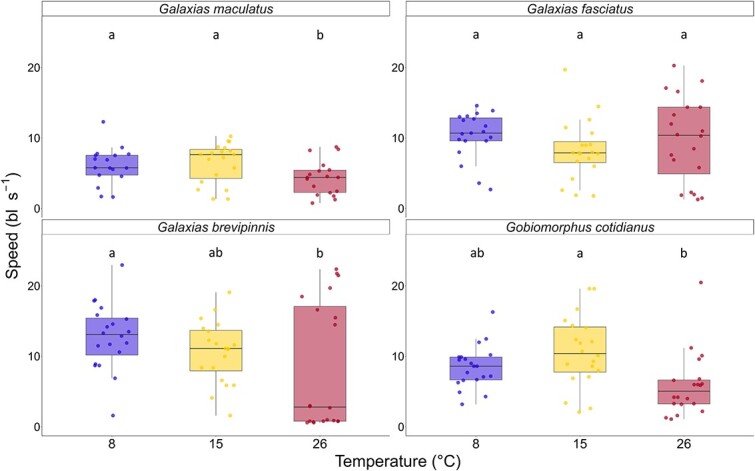
Boxplots of critical swimming speed for each temperature treatment (8, 15 and 26°C) for each species, measured in body lengths per second. Individual fish in each treatment group are represented by points. The centre bar of each box corresponds to the median, whilst the box outer edges indicate the interquartile range. The whiskers extend to 1.5 times the interquartile range. Within each plot, letters above the bars represent temperature treatments that are significantly different from one another (*P* < 0.05). Groups sharing the same letter are not significantly different (*P* > 0.05).

*Post hoc* pairwise comparisons using Tukey’s Honestly Significant Difference (HSD) revealed that critical swimming speed was reduced significantly in *G. maculatus*, *G. brevipinnis* and *G. cotidianus* at 26°C compared to the 8 or 15°C treatments (Table 3, Fig. 1). There was no significant difference in critical swimming speed at any of the three temperature treatments for *G. fasciatus*. There was also no statistically significant effect of fish length on critical swimming speed for any of the species; however, low *P*-values were recorded. The lack of significance may be a result of small sample size reducing statistical power or reflect the relatively narrow range of fish lengths tested.

**Table 3:** Summary of Type III analysis of covariance with Satterthwaite’s method comparing the difference between critical swimming speed with temperature and length for each species

**Species**	**Source**	**Sum Sq.**	**Mean Sq.**	**Num. d.f.**	**Den. d.f.**	**F value**	***P* (>F)**
*G. maculatus*	Temperature	46.2	23.12	2	50	3.43	0.013
	Length	20.5	20.53	1	50	3.05	0.087
*G. fasciatus*	Temperature	44.58	22.289	2	55	0.99	0.38
	Length	3.71	3.81	1	55	0.17	0.68
*G. brevipinnis*	Temperature	201.9	100.96	2	56	2.49	0.091
	Length	53.9	53.86	1	56	1.33	0.25
*G. cotidianus*	Temperature	233.2	116.6	2	56	6.32	0.003
	Length	3.3	3.35	1	56	0.18	0.67

Water temperature had a significant effect on *U_crit_* of *G. maculatus* (*P* = 0.01). Pairwise comparisons showed that mean *U_crit_* was statistically significantly different at 26°C (4.35 bl s^−1^, SD 2.44) when compared to both the 8 and 15°C treatments (6.11 bl s^−1^, SD 2.62 and 6.45 bl s^−1^, SD 2.85, respectively) (Table 3, Table 4, Fig. 1). There was no statistically significant difference in mean *U_crit_* between the 15 and 8°C treatments for *G. maculatus* (Table 4). The effect of fish length on *U_crit_* was not statistically significant at the predetermined significance threshold (*P* < 0.05); however, a low *P*-value was recorded (*P* = 0.09) (Table 3).

**Table 4:** Results of *post hoc* Tukey HSD tests for each species

**Species**	**Temperature comparison (°C)**	**Mean difference**	***P*-value**
*G. maculatus*	8–15	0.23	0.96
	8–26	−2	0.07
	15–26	−2.23	0.03
*G. fasciatus*	8–15	−2.06	0.37
	8–26	−0.54	0.93
	15–26	1.5	0.58
*G. brevipinnis*	8–15	−2.15	0.54
	8–26	−4.49	0.04
	15–26	−2.35	0.48
*G. cotidianus*	8–15	2.18	0.25
	8–26	−2.64	0.13
	15–26	−4.82	0.002

Water temperature had no statistically significant effect on *U_crit_* for *G. fasciatus* (*P* = 0.38) (8°C: 10.34 bl s^−1^, SD 3.31; 15°C: 8.28 bl s^−1^, SD 4.27; 26°C: 9.78 bl s^−1^, SD 6.03) (Table 3, Fig. 1). Pairwise comparisons showed no statistically significant difference between each of the three temperature treatments (Table 4, Fig. 1). The effect of fish length on *U_crit_* was not statistically significant (*P* = 0.68) (Table 3).

Water temperature had no statistically significant effect on *U_crit_* of *G. brevipinnis* at the 0.05 threshold (*P* = 0.09) (Table 3, Fig. 1). Pairwise comparison showed mean *U_crit_* was statistically significantly different at 26°C (8.29 bl s^−1^, SD 9.01) when compared to 8°C (12.79 bl s^−1^, SD 4.65) (Table 4, Fig. 1). There was no significant difference between the 15°C treatment (10.64 bl s^−1^, SD 4.39) and the 26°C, or between the 15 and the 8°C treatment (Table 4). Fish length had no statistically significant effect on critical swimming speed (*P* = 0.25) (Table 3).

Water temperature had a statistically significant effect on *U_crit_* of *G. cotidianus* (*P* = 0.003) (Table 3, Fig. 1). Pairwise comparisons showed mean *U_crit_* was statistically significantly different at 26°C (5.85 bl s^−1^, SD 4.47) when compared to 15°C (10.67 bl s^−1^, SD 5.04) (Table 4). There was no statistically significant effect of the 8°C treatment on *U_crit_* when compared to 15°C (8.5 bl s^−1^, SD 3.02 and 10.67 bl s^−1^, SD 5.04, respectively) or between the 8 and the 26°C treatment (Table 4). Fish length had no statistically significant effect on critical swimming speed of *G. cotidianus* (*P* = 0.67) (Table 3).

## Discussion

Our research sought to understand the effect of acute water temperature change on swimming performance of several different migratory fish species native to New Zealand, encompassing a range of physiological, morphological and life history traits. Critical swimming speeds for *G. maculatus, G. brevipinnis* and *G. cotidianus* were significantly reduced at warm water temperatures compared to temperate and/or cold temperature treatments. *Galaxias maculatus* and *G. cotidianus* had significantly reduced critical swimming speeds at 26°C when compared to 15°C. There was no observed difference in swimming ability between the 8 and 15°C treatments for either species. This corresponds with previous research by Bannon (2006), who showed that *G. maculatus* had reduced swimming performance at high temperatures. There was no observed difference in swimming ability between 26 and 8°C for *G. cotidianus* and *G. brevipinnis.*

At the highest water temperature, *G. brevipinnis* critical swimming speeds exhibited a bimodal distribution and a large variation of swimming performance amongst individuals, which was not observed at the other two temperatures. This may indicate an intraspecific effect of temperature on individuals, whereby some individuals have a higher thermal tolerance than others. Or this may be an indicator that 26°C is on the threshold of the upper thermal tolerance for this species (Taylor *et al.,* 1997; Norin and Clark, 2016). Previous research has shown that there are significant variations in swimming speeds both within and between populations of a species, due to variations in individual traits such as length, weight and metabolism (Crawford *et al.,* In Review; Beamish, 1978; Boily and Magnan, 2002; Ojanguren and Brana, 2003; Metcalfe *et al.,* 2016; Jones *et al.,* 2020). This is consistent with the range of metabolic rates that we observed in *G. maculatus* at both the temperate and high temperature treatments (Supplementary material: Table S1, Fig. S1).

Temperature effects on fish swimming performance typically follow a bell-shaped curve, with a peak in swimming performance at an optimum temperature range, and lower performance at temperature extremes (Brett and Brett, 1971; Beamish, 1978; McKenzie and Claireaux, 2010). This is often referred to as a thermal performance curve. In our study, we did not observe a significantly lower swimming performance at our low temperature treatment (i.e. 8°C) for any of our test species/life stages. The 8°C treatment falls within the normal range of what these species might experience in the wild, particularly *Galaxias brevipinnis* as they can penetrate further inland to higher elevations and may be adapted to cope with these cooler temperatures (McDowall, 1990). However, lower temperatures than we studied may elicit a negative response in swimming performance, for example, Bannon (2006) showed that the larval life stage of *G. maculatus* had significantly reduced swimming performance at 5°C.

Research has demonstrated that increasing temperatures come with increased energy costs for fish (Dickson *et al.,* 2002; McKenzie *et al.,* 2007; McKenzie and Claireaux, 2010). Higher temperature affects metabolic processes, consistently leading to an exponential increase in maintenance metabolism and standard metabolic rates across species, due to the energy demands of maintaining homeostasis (Brett and Brett, 1971; Fry, 1971; Taylor *et al.,* 1997). Temperature also affects MMR, which is the highest attainable level of aerobic metabolism (McKenzie and Claireaux, 2010). As temperatures rise, MMR typically increases and is strongly correlated to increased aerobic swimming ability due to increased energy fluxes and metabolic demands (Rome, 1990; Taylor *et al.,* 1997). Typically, as temperatures approach species’ upper thermal limits, MMR may plateau or even decrease. This decrease in MMR is attributed to the detrimental effects of high temperatures on convective oxygen transport and is correlated to a reduction in aerobic swimming ability (Jones, 1971; Taylor *et al.,* 1997). The upper thermal limit is species- and life stage-specific, whereby some species are more susceptible to high temperatures than others, leading to differences in species MMR and swimming ability (McKenzie and Claireaux, 2010). For instance, *Oncorhynchus mykiss* had both a reduction in aerobic swimming ability and a reduction in MMR at high temperatures, indicating that high temperatures significantly constrain aerobic swimming ability for this species (Taylor *et al.*, 1996). Conversely, *Dicentrarchus labrax* exhibited increasing MMR and increasing aerobic swimming ability at high temperatures, indicating that high temperatures enhance aerobic capacity (Claireaux *et al.,* 2006).

In our study, we observed both a decrease in critical swimming speed and a decrease in maximum metabolic rate in *G. maculatus* when exposed to a high-temperature treatment (26°C). Notably, *G. maculatus* prefers temperatures between 18 and 20°C, with a lethal limit recorded at 30.5°C (Richardson *et al.,* 1994). This suggests that 26°C approaches the upper limit of thermal tolerance for this species and likely the other Galaxiids studied. This is supported by Simons (1986), who proposed that Galaxiids should not be exposed to temperatures >26–27°C, as fish exhibited behavioural abnormalities and increased mortality beyond this range. If we consider 26°C as the upper thermal limit for Galaxiids, we expect both a decrease in swimming performance and maximum metabolic rate. At these elevated temperatures, at the upper end of thermal tolerance, fish are compelled to allocate more energy to oxygen uptake and routine maintenance (SMR), reducing aerobic capacity for other activities (Taylor *et al.,* 1997; Norin and Clark, 2016). This shift in energy allocation has been shown to result in reductions in critical swimming speeds in other species (Claireaux *et al.,* 2006; Norin and Clark, 2016).

Fish, particularly migratory species, frequently encounter temperature fluctuations, especially in freshwater systems as they migrate between habitats. This natural thermal heterogeneity is increasingly exacerbated by human alterations to freshwater environments (e.g. cold-water releases from dams, excessive abstraction, hot water discharges, urban stormwater runoff) (Caissie, 2006; Somers *et al.,* 2013; Parisi *et al.,* 2020; Pander *et al.,* 2024). Temperature during time of migration has been found to be a significant factor for migratory success and successful passage through instream structures (Haro *et al.,* 2004; Meixler *et al.,* 2009; Caudill *et al.,* 2013). Fish encountering higher temperatures due to instream structures often delay migration through these structures (Caudill *et al.,* 2013). When temperatures in fishways approach or exceed thermal limits, fish experience high stress levels. Multiple barriers with water temperature changes are expected to compound adverse effects on fish fitness, ultimately reducing migratory success due to increased energetic costs associated with migration. However, many models that assess fish passage barriers and barrier prioritization have not previously addressed the temperature effect on migrating fish (Meixler *et al.,* 2009). A new model put forward by Meixler (2021) highlights the importance of including temperature as a factor when assessing fish passage barriers and the need to understand the effects of temperature on fish swimming abilities to determine passage success.

Potential delays in migration become especially evident when we calculate the expected time for a fish to pass through a structure of a given length. For instance, considering the average length of culverts in New Zealand (20 m) and the average water velocity through a culvert (0.37 m s^−1^) (Franklin *et al.,* 2022; New Zealand Fish Passage Assessment Tool Database, https://fishpassage.niwa.co.nz/) along with the mean *U_crit_* of *G. maculatus* swimming at 15°C, we would anticipate the fish to pass through the culvert in ~197 s. Under the same conditions, but with the average *U_crit_* of *G. maculatus* swimming at 26°C, we would no longer expect them to successfully navigate the culvert due to the water speed exceeding *U_crit_* at this temperature, meaning they make negative progress overground. This reduction in migration ability is supported by Caudill *et al.* (2013), who found that a temperature gradient of >1°C across a fish ladder corresponded to changes in fish body temperature, indicating departure from acclimation temperature during passage.

Climate change, along with escalating habitat modification and land use alterations, will result in heightened discharge variability, elevated average stream temperatures, and shifting temperature and runoff patterns (Paul and Meyer, 2001; Morgan and Cushman, 2005). Additionally, it will amplify the severity and frequency of droughts and floods (Milly *et al.,* 2005; Alcamo *et al.,* 2007). All these factors will lead to greater extremes in thermal heterogeneity that may be hard to predict. Restoring connectivity is one of the six actions identified in the Emergency Recovery Plan for global freshwater biodiversity (Tickner *et al.,* 2020). To future-proof our efforts to restore connectivity, it is important to consider temperature effects on fish dispersal and swimming ability. If maximum swimming speeds decrease as temperature increases, then to future-proof our designs for a constantly changing environment, we need to design fish passes for lower swimming speeds than have previously been measured. This reduction in swimming ability could culminate in reductions in species’ dispersal abilities, with implications for their ongoing ability to access suitable habitats.

Previous research has shown that temperature effects on fish are species-specific. For example, some studies have demonstrated that cold water (more so than warm water) has significant negative impacts on temperate and tropical fish swimming performance (Johnston and Dunn, 1987; Fangue *et al.,* 2015; Parisi *et al.,* 2020). Muhawenimana *et al.* (2021) observed that in a temperate location, warmer waters improved invasive species’ swimming performance. This species-specific response to temperature is highlighted by our research. There was no observed effect of water temperature on *G. fasciatus* swimming performance, but there was a significant reduction in swimming performance at high temperatures for both *G. maculatus* and *G. cotidianus*. In *G. brevipinnis*, we observed a bimodal distribution in swimming performance at high temperature, indicating that temperature effects also vary within a species.

Crawford *et al.* (In Review) demonstrated the importance of accounting for individual variation in swimming ability when developing water velocity design criteria for fish passage. Here we have demonstrated that water temperature adds an additional dimension of variability that should be accounted for to future-proof fish passage design criteria.

## Supplementary Material

Web_Material_coae047

## Data Availability

Critical swimming speed and endurance data will be available via the Zenodo digital repository 10.5281/zenodo.10086037
