# Supplementary material for: High water temperature significantly influences swimming performance of New Zealand migratory species
Source: Conserv Physiol. 2024 Jul 31;12(1):coae047. doi: 10.1093/conphys/coae047 (PMC11289306; doi:10.1093/conphys/coae047)
Supplement: Web_Material_coae047 [file web_material_coae047.pdf]

Supplementary material

Maximum metabolic rate

Galaxias maculatus was used as our model species for measuring maximum metabolic rate (MMR) as it exhibited the lowest U_{crit} (bl s^{-1}) when compared to a range of native species (Crawford et al, in review). Immediately after fatigue, post-exercise maximum metabolic rates of *Galaxias maculatus* individuals were measured using a closed circuit respirometry unit, following the methods of Parisi et al. (2020). Fish length and weight were recorded before (MMR) trials. The closed circuit respirometry unit consisted of a 115 mL plastic container connected to a recirculating flow system. The respirometer was placed in a water bath held at the trial temperature (either 15°C or 26°C). Maximum metabolic rate was measured for 16 *Galaxias maculatus* at 15°C and 20 *Galaxias maculatus* at 26°C. The respirometer was connected to a Loligo System oxygen sensor, which measured oxygen concentration (% air saturation) every minute for 15 minutes. The metabolic rate of the fish was calculated using the following equation from Schurmann & Steffensen (1997):

$$MMR = -1 \times \Delta O_2 \times V \text{ x } M_{fish}^{-1} \times \beta O_2$$

Where: ΔO_2 is percent change in air saturation per hour); V is volume of the respirometer in litres, without the mass of the fish (assuming a density of 1 g ml^{-1}); M_{fish} is the mass of the fish in grams; βO_2 is the solubility of oxygen (mg L^{-1}) at the trial temperature (10.08 mg L^{-1} at 15°C and 8.11 mg L^{-1} at 26°C) (YSI, 2019).

An ANCOVA was used to test for the effects of water temperature on oxygen consumption in *Galaxias maculatus*. Maximum metabolic rate calculated as oxygen consumption (mg O_2 g^{-1} h^{-1}) was the response variable, with water temperature as the

categorical predictor variables and length, weight, and critical swimming speed as the continuous predictor variables. To assess model fit, a Likelihood Ratio Test was used, starting with a fully saturated model including interactions. The interaction between water temperature and weight was statistically significant and kept in the model. Average length and critical swimming speed were not statistically significant but were kept in the model as these variables are known to influence MMR (Norin & Clark, 2016).

Results

Maximum metabolic rate was significantly reduced (p= 0.006) in *Galaxias maculatus* at 26°C (3.19 mg O_2 g^{-1} h^{-1}, SD 2.19) when compared to 15°C (8.54 mg O_2 g^{-1} h^{-1}, SD 8.43) (Table S1, Figure S1). There was greater variation in oxygen consumption at 15°C, with maximum metabolic rates ranging from 0.31 to 15.54 mg O_2 g^{-1} h^{-1} (with an outlier at 34.76mg O_2 g^{-1} h^{-1}) and a larger standard deviation, when compared to the 26°C treatment (ranging from 0.23 to 7.08 mg O_2 g^{-1} h^{-1}). We assume there was a methodological issue with the outlier, as the fish had an average U_{crit} (5.1 bl s^{-1}) and was of average length. When the outlier was removed from the analysis, MMR at 15°C was still significantly higher than 26°C. Fish weight was statistically significant (p= 0.034) and so was the interaction between water temperature and fish weight (p=0.01).

Table S1: Summary of Type III analysis of covariance with Satterthwaite's method comparing the difference between *Galaxias maculatus* post-exercise oxygen consumption (mg O_2 g^{-1} h^{-1}) at 15°C and 26°C.

**Source**	**Sum Sq.**	**Mean Sq.**	**Num. d.f.**	**Den. d.f.**	**F value**	**P (>F)**
Temperature	236.5	236.49	1	29	8.63	0.006
Weight	135.5	135.46	1	29	4.94	0.034
Length	19.8	19.78	1	29	0.72	0.402
Critical swimming speed	8.3	8.34	1	29	0.30	0.585
Temperature:Weight	205.5	205.55	1	29	7.50	0.010

Figure S1:

Boxplots of post-exercise maximum metabolic rate (mg O_2 g^{-1} h^{-1}) compared to each temperature treatment (15°C and 26°C) for *Galaxias maculatus*.

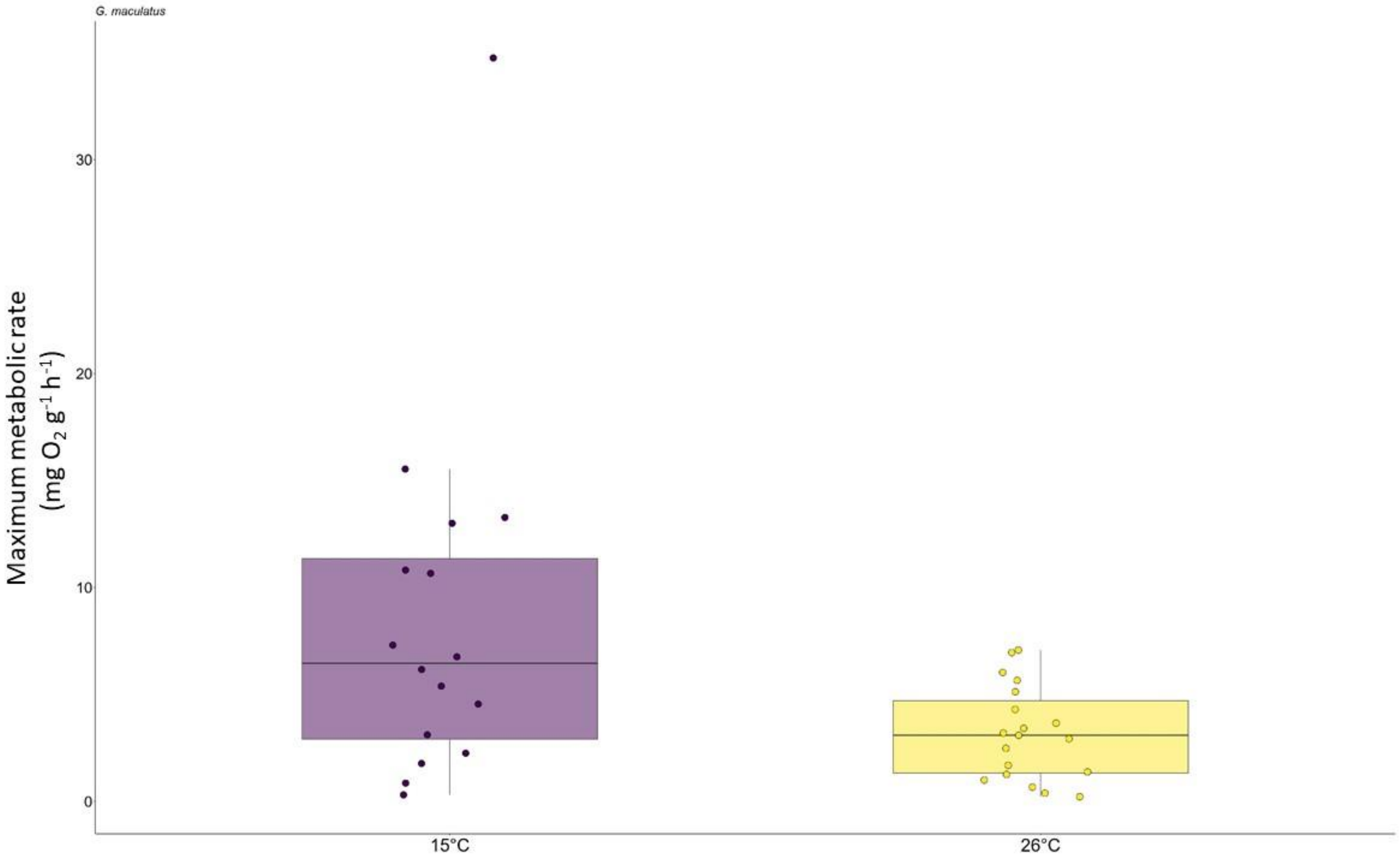

G. maculatus
Maximum metabolic rate
(mg O2 g-1 h-1)
30
20
10
0
15°C
26°C
